# Assessing Differential Expression in Two-Color Microarrays: A Resampling-Based Empirical Bayes Approach

**DOI:** 10.1371/journal.pone.0080099

**Published:** 2013-11-27

**Authors:** Dongmei Li, Marc A. Le Pape, Nisha I. Parikh, Will X. Chen, Timothy D. Dye

**Affiliations:** 1 Office of Public Health Studies, John A. Burns School of Medicine, University of Hawaii at Manoa, Honolulu, Hawaii, United States of America; 2 John A. Burns School of Medicine, University of Hawaii, Honolulu, Hawaii, United States of America; 3 Cardiovascular Division, The Queens Medical Center, Honolulu, Hawaii, United States of America; University of Bonn, Bonn-Aachen International Center for IT, Germany

## Abstract

Microarrays are widely used for examining differential gene expression, identifying single nucleotide polymorphisms, and detecting methylation loci. Multiple testing methods in microarray data analysis aim at controlling both Type I and Type II error rates; however, real microarray data do not always fit their distribution assumptions. Smyth's ubiquitous parametric method, for example, inadequately accommodates violations of normality assumptions, resulting in inflated Type I error rates. The Significance Analysis of Microarrays, another widely used microarray data analysis method, is based on a permutation test and is robust to non-normally distributed data; however, the Significance Analysis of Microarrays method fold change criteria are problematic, and can critically alter the conclusion of a study, as a result of compositional changes of the control data set in the analysis. We propose a novel approach, combining resampling with empirical Bayes methods: the Resampling-based empirical Bayes Methods. This approach not only reduces false discovery rates for non-normally distributed microarray data, but it is also impervious to fold change threshold since no control data set selection is needed. Through simulation studies, sensitivities, specificities, total rejections, and false discovery rates are compared across the Smyth's parametric method, the Significance Analysis of Microarrays, and the Resampling-based empirical Bayes Methods. Differences in false discovery rates controls between each approach are illustrated through a preterm delivery methylation study. The results show that the Resampling-based empirical Bayes Methods offer significantly higher specificity and lower false discovery rates compared to Smyth's parametric method when data are not normally distributed. The Resampling-based empirical Bayes Methods also offers higher statistical power than the Significance Analysis of Microarrays method when the proportion of significantly differentially expressed genes is large for both normally and non-normally distributed data. Finally, the Resampling-based empirical Bayes Methods are generalizable to next generation sequencing RNA-seq data analysis.

## Introduction

Microarray technology is widely used to examine the activity level of thousands of genes simultaneously in human cells to better understand differential gene activation across diseases, such as heart diseases, infectious diseases, mental illness, and health disparities across ethnic groups. For example, DNA microarrays are widely used for DNA methylation studies - which are increasingly recognized as an important biological factor in ethnicity-based health disparities. A recent study shows that significantly different DNA methylation levels at birth, between Caucasians and African Americans, partially explain the incidence rates differential of specific cancers between ethnicities [1].

DNA methylation experiments typically use single channel or two-color microarrays for detecting DNA methylation differences between different groups. Smyth's parametric model (PM) [2], one of the most frequently used and most powerful models for two-color micoarray data analysis, is available through the lmFit and eBayes function in the open source Bioconductor/R software's limma package. The traditional approach to microarray analysis is the ordinary 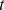-statistic [3]. However, a large 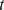-statistic may result from an unrealistically small standard deviation. Thus, genes with small sample variances are more likely to have large 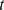-statistics even when they are not differentially expressed. Both Tusher et al. [4] and Efron et al. [5] modified the ordinary 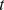-statistic to have penalized 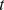-statistics by adding a penalty to the standard deviation. The penalty in Tusher's method is chosen to minimize the sample variation coefficient, while Efron et al. chose the penalty as the 90th percentile of the sample standard deviation values. In simulation studies, Lönnstedt and Speed [6] showed that both forms of penalized 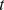-statistics were far superior to the ordinary 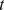-statistic for selecting differentially expressed genes. They further modified the penalized 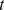-statistics through a parametric empirical Bayes approach using a simple mixture of normal models and a conjugate prior, and showed that their empirical Bayes method had both lower Type I error rates and Type II error rates compared to the penalized 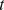-statistics.

Smyth developed the hierarchical model of Lönnstedt and Speed into a practical approach for general microarray experiments with arbitrary number of treatments and RNA samples using a moderated 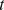-statistic that follow a 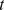-distribution with augmented degrees of freedom. Smyth also showed in simulation studies that the moderated 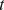-statistic has the largest area under the Receiver Operating Curve, with both lower Type I and lower Type II error rates compared to ordinary 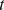-statistics, Efron's penalized 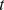-statistics, and Lönnstedt and Speed's empirical Bayes statistic. However, Smyth's method calculates *p*-values based on the 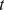-distribution, which could generate incorrect *p*-values for non-normally distributed microarray data. Another widely used microarray data analysis method, Significance Analysis of Microarrays (SAM) [4], is based on permutation test and robust to violations of the 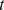-distribution. However, fold change threshold selection in the SAM method is problematic as different fold change criteria can critically alter the conclusions of a study, resulting from compositional changes of the control data set in the analysis [7]. As such, to reduce false discovery rates for non-normally distributed microarray data, we propose a novel approach combining resampling with empirical Bayes methods: Resampling-based empirical Bayes Methods (RBMs). This approach is impervious to fold change criteria as no control data set selection is needed; furthermore, this novel approach is generalizable to next generation sequencing RNA-seq data analysis.

## Methods

### Ethics Statement

The data used in this paper to argue the false discovery controls of PM and RBMs were collected in accordance with the University of Hawaii IRB CHS #20067 terms of approval for a placental DNA methylation study deemed exempt from federal regulations pertaining to the protection of human research participants. Authority for exemption is documented in Title 45, Code of Federal Regulations, Part 46. The methylation microarray data have been deposited in NCBI's Gene Expression Omnibus [8] and are accessible through GEO Series accession number GSE49504 (http://www.ncbi.nlm.nih.gov/geo/query/acc.cgi?acc=GSE49504).

### FDR, Sensitivity, and Specificity

Suppose we are testing *m* null hypotheses simultaneously in a DNA microarray study. Among the *m* hypotheses, 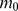 of the hypotheses are true. For any multiple testing procedure that reject 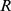 null hypotheses out of *m* null hypotheses, we use 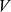 to denote the number of falsely rejected true null hypotheses (false discoveries) among 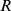 rejections, and use 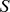 to denote the number of true rejections among 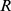 rejections (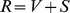). Table 1 shows the possible outcomes when testing *m* null hypotheses simultaneously.

**Table 1:** Possible outcomes of testing *m* null hypotheses.

	Numbernot rejected	Numberrejected	Total
True null hypotheses	*U*	*V*	*m*_0_
Non-true null hypotheses	*T*	*S*	*m*-*m*_0_
Total	*m*-*R*	*R*	*m*

*m* is the total number of null hypotheses.

The framework of false discovery rate (FDR) was proposed by Soric [9] for quantifying the statistical significance based on the rate of false discoveries. The formal definition of FDR was proposed by Benjamini and Hochberg [10] as:
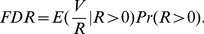
(1)


For a discovery-based microarray study, FDR is generally recognized as an appropriate multiple testing error rate with 5% as the most commonly used cutoff value. When comparing different methods for microarray data analysis, high sensitivity and specificity are often desired properties of a good microarray analysis method. Sensitivity is defined as the probability of rejecting a non-true null hypothesis, while specificity is defined as the probability of failing to reject a true null hypothesis. The sensitivity and specificity of a multiple testing procedure can be calculated as follows:
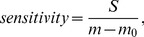
(2)

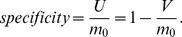
(3)


Sensitivity relates to a test's ability to identify positive results (giving the proportion of true positives) and is also a definition of power in multiple testing. A test with a high sensitivity has a low type II error rate and high power.

Specificity relates to a test's ability to identify negative results. A test with high specificity has a low type I error rate which is important to control for.

### Resampling-based multiple testing procedures

Resampling-based multiple testing procedures are widely used in genomic studies to identify differential gene expression and to conduct genome-wide association studies (GWAS), particularly when the distribution of test statistics is non-normally distributed or unknown. Meanwhile, resampling-based multiple testing procedures can also take into account dependence among p-values or test statistics. Commonly used resampling techniques include permutation tests and bootstrap methods.

A permutation test is a type of non-parametric statistical significance test in which the test statistics' distribution under the null hypothesis is constructed by calculating all possible values or a concrete number of test statistics (usually 1000 or above) from permuted observations under the null hypothesis. The theory of permutation tests is based work done by Fisher and Pitman in the 1930s [11]. Permutation tests are distribution-free, and can provide exact *p*-values even when the sample size is small. Westfall and Young [12] elaborated upon multiple testing procedures using the permutation test, and it has been shown that the permutation test has a strong control of multiple testing error rate under the marginal-determining-joint condition [13].

The bootstrap method, first introduced by Efron [14], and further discussed by Efron and Tibshirani [15], is a way of approximating the sampling distribution from just one sample. Instead of taking many simple random samples from the population to find a sample statistic's sampling distribution, the bootstrap method repeatedly resamples with replacement from one random sample. Efron [14] showed that the bootstrap method is an asymptotically unbiased estimate for the variance of a sample median, and for error rates in a linear discrimination problem - outperforming cross-validation. Freedman [16] conclusively showed that the bootstrap approximation to the distribution of least square estimates is valid. Finally, Hall [17] showed that the bootstrap method's reduction of error coverage probability, from 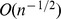 to 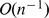, makes the bootstrap method one order of magnitude more accurate than the delta method. The p-values computed by the bootstrap method are less exact than the p-values obtained from the permutation method. It has been proved that the p-values estimated by the bootstrap method are asymptotically convergent to the true p-values [18].

### Significance Analysis of Microarrays (SAM) procedure

The Significance Analysis of Microarrays (SAM) was first introduced by Tusher et al. [4] to identify statistically significant differences in gene expression by assimilating a set of gene-specific *t* tests. In SAM, each gene is assigned a score on the basis of its difference in gene expression relative to the standard deviation of repeated measurements for that gene. A scatter plot of the observed relative difference, versus the expected relative difference estimated by the permutation method, is then used to select statistically significant genes based on a pre-determined threshold.

The SAM procedure can be summarized as follows.

- Compute a test statistic 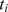 for each gene *i* (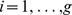).
- Compute order statistics 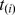 such that 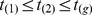.
- Perform *B* permutations of the responses/covariates 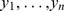. For each permutation *b*, compute the permuted test statistics 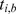 and the corresponding order statistics 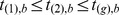.
- From the *B* permutations, estimate the expected values of order statistics by 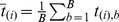.
- Form a quantile-quantile (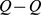) plot (SAM plot) of the observed 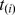 versus the expected 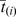.
- For a given threshold Δ, starting at the origin, and moving up to find the first 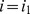 such that 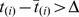. All genes past 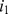 are called significant positives. Similarly, starting at the origin, and moving down to the left, find the first 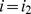 such that 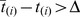. All genes past 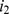 are called significant negatives. Define the upper cut point 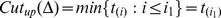, and the lower cut point 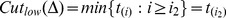.
- For a given threshold, the expected number of false rejections 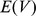 is estimated by computing the number of genes with 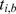 above 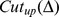 or below 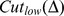 for each of the *B* permutations, and averaging the numbers over *B* permutations.
- A threshold Δ is chosen to control the 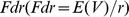 under the complete null hypothesis, at an acceptable nominal level.

In our simulation studies, the SAM procedure is implemented through the sam function in the Bioconductor's siggenes package.

### Linear models and empirical Bayes method (PM)

**Figure 1:**
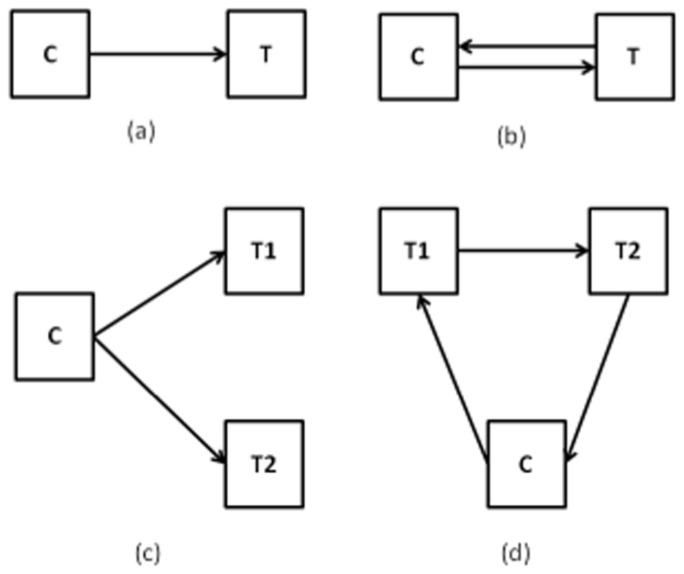
Commonly used experimental design for two color micoarrays.

In general, let 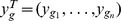 denote the log-ratios of two-color dye intensities or log-intensities for single color data which have been suitably normalized in a microarray experiment. The log-ratios of the two-color intensities or log-intensities for single color data can be expressed in a linear model format as follows:
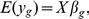
(4)where *X* is a design matrix of full column rank and 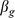 is a coefficient vector. The commonly used designs of a two-color microarray experiment described in Kerr and Churchill [19] are displayed in Figure 1. Each rectangle represents a DNA/RNA sample with C denoting control and T denoting treatment samples. Each arrow denotes a microarray. The DNA/RNA sample on the left of the arrow will be dyed with cy3 (green dye) and the RNA sample on the right of the arrow will be dyed with cy5 (red dye). Design (a) in Figure 1 examines whether the log2 differences of red dye intensities (T) and green dye intensities (C) between treatment and control samples are equal to 0. Design (b) swaps the two dyes and generates two log2 differences, T-C and C-T. The design matrix *X* for design (b) is
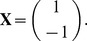



The design matrix X for design (c) and design (d) could be as follows:
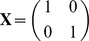
for design (c), and
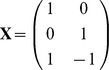
for design (d).

Different from two color microarrays, single color microarrays usually have a single expression value for each gene and each array. The design matrix for single color microarrays can be formed exactly as in classic linear model settings based on biological factors in microarray experiments.

We assume 
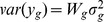
 and 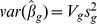. 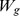 is a known non-negative definite weight matrix. 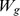 may contain diagonal weights of zero for missing values in 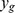. 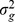 is the unknown error variance for 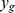. 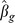 is the estimated coefficient vector. 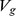 is a positive definite matrix not depending on 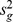 which is the residual variance for *g*th gene. Let 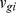 be the *j*th diagonal element of 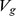. Smyth [2] assumes the following distributional assumptions:
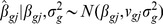
(5)and
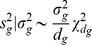
(6)where 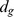 is the residual degrees of freedom for the linear model for gene *g*. The ordinary *t*-statistic under these assumptions is
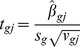
(7)which follows an approximate 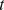-distribution on 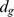 degrees of freedom.

A prior distribution on 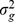 is assumed as equation (8) with prior estimator 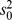 and 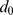 degrees of freedom estimated from the data by equating empirical to expected values for the first two moments of 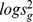, which is used because of its finite property for any degrees of freedom and an even more nearly normal distribution than 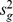, so that moment estimation is likely to be more efficient.
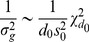
(8)


The posterior mean of 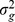 given 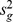 is
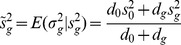
(9)


Then the moderated *t*-statistic, based on a hybrid classical/Bayes approach, is defined by:
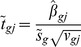
(10)


The *p*-value for testing 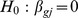 based on the moderated 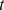-statistic can be calculated from 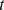 distribution with 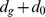 degrees of freedom. Appropriate quadratic forms of the moderated 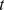-statistics follow 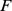-distributions and can be used to test hypotheses about any set of contrasts simultaneously. Smyth's method is available through the limma package in Bioconductor, and is widely used for two-color microarray data analysis.

### Resampling and empirical Bayes methods (RBMs)

To carry out the permutation/bootstrap test based on the moderated t-statistics proposed by Smyth [2], we proceed as follows:

- Compute the moderated 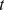-statistics 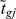 based on the observed data set for each gene *g*.
- Permute/bootstrap the original data in a way that matches the null hypothesis to get permuted/bootstraped resamples, and construct the reference distribution using the moderated 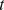-statistics or 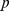-values calculated from permuted/bootstrapped resamples.
- Calculate the critical value of a level *α* test based on the upper *α* percentile of the reference distribution, or obtain the *p*-value by computing the proportion of permutation/bootstrap test statistics or *p*-values that are as extreme as, or more extreme than, the observed moderated 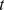-statistic or *p*-value.

The *p*-values for the *p*-value based permutation/bootstrap methods are calculated according to the following formula:
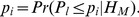
(11)


Similarly, the p-values for the test statistics-based permutation/bootstrap methods are calculated from the following formula:
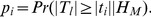
(12)


In the above formulas, 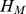 denotes the complete null hypothesis and 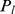 denotes the random variable for the raw *p*-value of the *l*th hypothesis.

Depending on the resampling method (either permutation or bootstrap) and the *p*-value calculation method (either test statistics or *p*-values), four RBM methods are proposed: RBM test statistic based permutation method (TSBP); RBM test statistic based bootstrap method (TSBB); RBM *p*-value based permutation method (PBP); RBM *p*-value based bootstrap method (PBB). Both the PM and the RBMs follow the empirical Bayes approach proposed by Efron et al. [5], thus controlling for false discovery rates in the data analysis.

### Simulation data sets

In our simulation studies, each data set includes 1000 independently generated samples of two groups of equal sample size of 4, 6, 12, 24, and 48. The sample sizes of 4 and 6 represent small sample size scenarios, 12 and 24 represent medium sample size scenarios, and 48 represents large sample size scenarios. The total number of genes (*m*) is set to be 500 with the fraction of differentially expressed genes (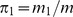) equal to 10%, 25%, 50%, 75%, and 90% to cover all possible scenarios. In the two-groups comparisons, the gene expression level on log2 scale is generated randomly, either from a multivariate normal distribution with 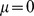 and 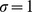, or from a multivariate log normal distribution with 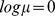 and 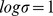, or from a mixed normal distribution (80% of the data follow a normal distribution with 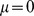 and 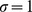, and 20% of the data follow a normal distribution with 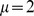 and 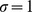), with random correlations between genes to mimic the correlations in real microarray data. Mean differences between groups are set to be 1, and standard deviations are randomly generated from a scaled chi-square distribution with 4 degrees of freedom. The number of permutation/bootstrap is set at 1000. In our simulation study, three reasons led us to choose 1000 permutations as the optimal number of permutations. The first reason was to standardize to the default number of permutations used in most statistical software packages such as Bioconductor and IBM SPSS. A second reason for selecting 1000 permutations was that a larger number of permutations was originally used in our simulation study with no significantly different results. Indeed, with 1000 permutations the smallest possible *p*-value is 0.001, and the uncertainty near *p* = 0.05 is about 1%; as our approach already controled for FDR and no further multiplicity adjustment was needed, 1000 permutations were deemed sufficient. A third and final reason lied with reducing computational load and fostering computational efficiency. The significance level was set at 5%. The R codes for our resampling and empirical Bayes methods are publicly available from http://www.hawaii.edu/publichealth/faculty/profile/li.html.

## Results

Simulation studies were conducted to compare the sensitivity, specificity, total rejection, and false discovery rate across the PM, the SAM, and the RBMs. The simulation studies include situations for both normally distributed and non-normally (log normally and mixed normally) distributed miroarray data.

### Simulation results from normally distributed data

**Figure 2:**
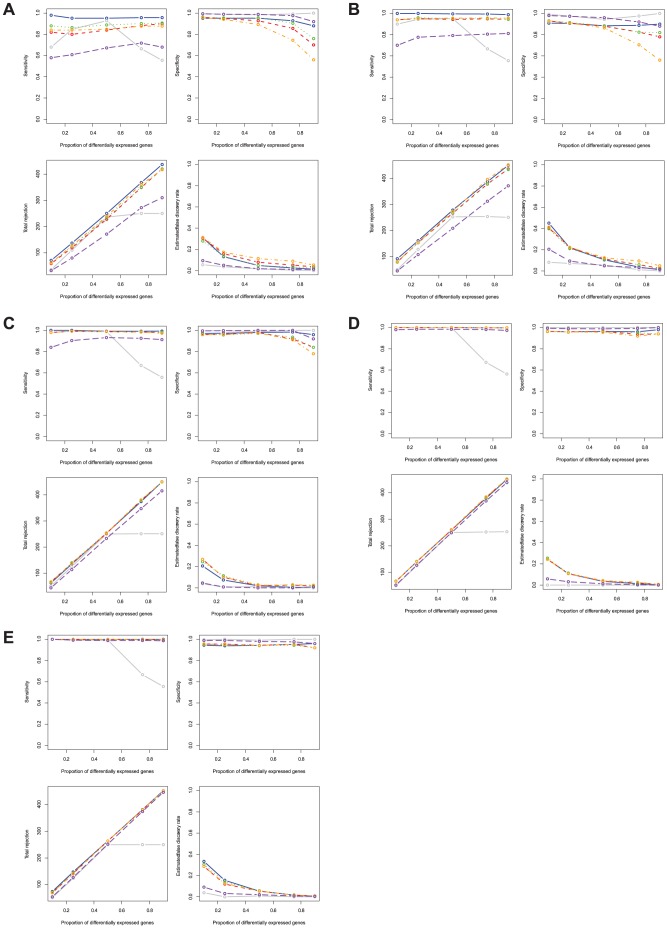
Sensitivity, specificity, total rejection, and estimated false discovery rate comparisons between the RBMs and the PM for normal distributed gene expression data. Blue: PM; Grey: SAM; Red: RBM test statistic based permutation method; Orange: RBM 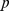-value based permutation method; Green: RBM test statistic based bootstrap method; Purple: RBM 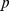-value based bootstrap method. Figure 2a: sample size *n* = 4 in each group; Figure 2b: sample size *n* = 6 in each group; Figure 2c: sample size *n* = 12 in each group; Figure 2d: sample size *n* = 24 in each group; Figure 2e: sample size *n* = 48 in each group.

In terms of sensitivity (power), the PM shows very high sensitivity across all sample sizes and higher sensitivity than all other methods - even when sample size is small, e.g., 4 or 6 in each group (Figure 2a, b and Table 2). Both the SAM and the PBB has lower sensitivity compared to other methods for small sample sizes. However, the sensitivity improves significantly as sample size in each group increases for the PBB method, but not for the SAM method. The SAM method shows low sensitivity when the proportion of differentially expressed genes is over 50% - regardless of sample size (Figure 2 and Table 2). All other RBM methods show good sensitivity levels, comparable to the PM method when 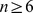 in each group (Figure 2b, 2c, 2d, 2e and Table 2).

**Table 2:** Comparison of sensitivities for all six methods.

Distribution	*n*	*π*_1_	PM	SAM	TSBP	TSBB	PBP	PBB
Normal	*n* = 4	0.10	0.980	0.680	0.820	0.880	0.840	0.580
		0.25	0.952	0.848	0.800	0.864	0.840	0.608
		0.50	0.952	0.928	0.840	0.888	0.848	0.672
		0.75	0.957	0.664	0.883	0.901	0.877	0.717
		0.90	0.958	0.556	0.900	0.907	0.878	0.680
	*n = 6*	0.10	1.000	0.900	0.940	0.940	0.940	0.700
		0.25	1.000	0.944	0.952	0.960	0.952	0.776
		0.50	0.996	0.952	0.944	0.952	0.956	0.792
		0.75	0.995	0.667	0.949	0.952	0.957	0.805
		0.90	0.989	0.556	0.944	0.944	0.956	0.811
	*n* = 12	0.10	1.000	0.980	0.980	0.980	0.980	0.840
		0.25	1.000	0.992	0.992	0.992	0.992	0.904
		0.50	0.992	0.988	0.988	0.988	0.988	0.932
		0.75	0.992	0.669	0.984	0.987	0.984	0.925
		0.90	0.993	0.558	0.980	0.982	0.973	0.913
	*n* = 24	0.10	1.000	1.000	1.000	1.000	1.000	0.980
		0.25	1.000	1.000	1.000	1.000	1.000	0.984
		0.50	1.000	1.000	1.000	1.000	1.000	0.984
		0.75	0.997	0.672	0.997	0.997	0.997	0.981
		0.90	0.998	0.562	0.998	0.998	0.998	0.973
	*n* = 48	0.10	1.000	1.000	1.000	1.000	1.000	1.000
		0.25	1.000	0.992	1.000	0.992	1.000	0.992
		0.50	1.000	0.992	1.000	0.996	1.000	0.988
		0.75	1.000	0.667	0.997	0.995	0.997	0.989
		0.90	1.000	0.556	0.996	0.996	0.996	0.987
Log Normal	*n* = 4	0.10	0.960	0.520	0.780	0.820	0.780	0.480
		0.25	0.944	0.768	0.784	0.808	0.792	0.544
		0.50	0.948	0.884	0.792	0.820	0.796	0.592
		0.75	0.949	0.664	0.819	0.840	0.829	0.608
		0.90	0.951	0.556	0.844	0.860	0.829	0.602
	*n* = 6	0.10	0.960	0.840	0.880	0.880	0.900	0.720
		0.25	0.984	0.896	0.920	0.912	0.936	0.672
		0.50	0.984	0.916	0.916	0.920	0.928	0.704
		0.75	0.984	0.667	0.907	0.912	0.912	0.723
		0.90	0.987	0.556	0.909	0.918	0.909	0.727
	*n* = 12	0.10	1.000	0.900	0.900	0.900	0.900	0.780
		0.25	1.000	0.968	0.944	0.944	0.944	0.840
		0.50	1.000	0.968	0.952	0.952	0.952	0.844
		0.75	0.997	0.669	0.952	0.955	0.952	0.864
		0.90	0.998	0.558	0.947	0.949	0.947	0.860
	*n* = 24	0.10	1.000	0.960	0.980	0.980	0.980	0.940
		0.25	1.000	0.976	0.976	0.976	0.976	0.928
		0.50	1.000	0.988	0.984	0.984	0.984	0.916
		0.75	1.000	0.672	0.987	0.987	0.987	0.923
		0.90	1.000	0.560	0.989	0.989	0.982	0.918
	*n* = 48	0.10	1.000	0.980	1.000	1.000	1.000	0.980
		0.25	1.000	0.984	0.992	0.992	0.992	0.968
		0.50	0.996	0.976	0.980	0.980	0.980	0.952
		0.75	0.997	0.667	0.981	0.981	0.981	0.949
		0.90	0.998	0.556	0.982	0.982	0.982	0.953
Mixed Normal	*n* = 4	0.10	0.920	0.820	0.900	0.920	0.880	0.840
		0.25	0.896	0.912	0.864	0.912	0.848	0.816
		0.50	0.932	0.960	0.900	0.936	0.900	0.860
		0.75	0.933	0.667	0.923	0.939	0.920	0.864
		0.90	0.927	0.556	0.940	0.942	0.931	0.853
	*n* = 6	0.10	0.940	0.920	0.940	0.940	0.940	0.920
		0.25	0.944	0.944	0.944	0.944	0.944	0.928
		0.50	0.964	0.980	0.964	0.964	0.964	0.936
		0.75	0.973	0.667	0.973	0.973	0.973	0.947
		0.90	0.967	0.556	0.962	0.967	0.962	0.944
	*n* = 12	0.10	1.000	1.000	1.000	1.000	1.000	1.000
		0.25	1.000	0.984	1.000	1.000	1.000	0.984
		0.50	0.992	0.996	0.996	0.996	0.996	0.984
		0.75	0.992	0.669	0.992	0.995	0.992	0.987
		0.90	0.993	0.558	0.991	0.991	0.991	0.982
	*n* = 24	0.10	1.000	1.000	1.000	1.000	1.000	1.000
		0.25	1.000	0.992	1.000	1.000	1.000	1.000
		0.50	0.996	1.000	1.000	1.000	1.000	0.996
		0.75	0.997	0.675	1.000	1.000	1.000	0.997
		0.90	0.998	0.562	1.000	1.000	1.000	0.996
	*n* = 48	0.10	1.000	1.000	1.000	1.000	1.000	1.000
		0.25	1.000	1.000	1.000	1.000	1.000	1.000
		0.50	1.000	1.000	1.000	1.000	1.000	0.996
		0.75	0.997	0.669	0.997	0.997	0.997	0.995
		0.90	0.998	0.558	0.998	0.998	0.998	0.996

*π*_1_: Proportion of differentially expressed genes.

TSBP: RBM Test statistic based permutation method.

TSBB: RBM Test statistic based bootstrap method.

PBP: RBM *p*-value based permutation method.

PBB: RBM *p*-value based bootstrap method.

All methods show comparable specificity when sample size is large (Figure 2c, 2d, and 2e). Both the PBB and the SAM methods show slightly higher specificity than the PM method even when sample size is small (Figure 2a and 2b). Other RBMs perform similarly to the PM when the proportion of differentially expressed genes is less than 50%, and sample size is small.

The number of total rejections for all methods shows similar trends as sensitivity (Figure 2). The RBMs have a slightly lower number of total rejections compared to the PM. The SAM has a comparable number of total rejections as the RBMs when the proportion of differentially expressed genes are less than 50%. As expected, the SAM has a lower number of total rejections due to its lower sensitivity compared to all other methods when the proportion of differentially expressed genes are over 50% across all sample sizes.

For false discovery rates control, the SAM method has the most conservative control rate among all methods compared for all sample sizes (Table 3). The conservativeness of the SAM method slightly increases with sample size. Both the SAM and the PBB methods have much lower estimated false discovery rates compared to the PM and other RBM methods when the proportion of differentially expressed genes are over 50% across sample sizes (Figure 2).

**Table 3:** Comparison of estimated false discovery rates for all six methods.

Distribution	*n*	*π*_1_	PM	SAM	TSBP	TSBB	PBP	PBB
Normal	*n = 4*	0.10	0.310	0.056	0.305	0.279	0.311	0.094
		0.25	0.131	0.036	0.160	0.136	0.173	0.050
		0.50	0.048	0.017	0.079	0.047	0.113	0.018
		0.75	0.025	0.004	0.052	0.034	0.089	0.011
		0.90	0.014	0.000	0.036	0.029	0.053	0.013
	*n* = 6	0.10	0.451	0.082	0.405	0.397	0.413	0.205
		0.25	0.219	0.071	0.222	0.211	0.222	0.094
		0.50	0.104	0.056	0.113	0.109	0.125	0.048
		0.75	0.036	0.012	0.058	0.058	0.093	0.032
		0.90	0.011	0.000	0.025	0.021	0.049	0.016
	*n* = 12	0.10	0.207	0.039	0.269	0.246	0.269	0.046
		0.25	0.074	0.008	0.101	0.114	0.101	0.009
		0.50	0.020	0.012	0.020	0.028	0.024	0.000
		0.75	0.005	0.000	0.026	0.021	0.029	0.000
		0.90	0.005	0.000	0.018	0.018	0.025	0.010
	*n* = 24	0.10	0.243	0.000	0.243	0.254	0.243	0.058
		0.25	0.107	0.000	0.107	0.114	0.107	0.032
		0.50	0.035	0.000	0.035	0.039	0.042	0.012
		0.75	0.013	0.000	0.021	0.016	0.026	0.003
		0.90	0.002	0.000	0.007	0.007	0.007	0.000
	n = 48	0.10	0.333	0.038	0.286	0.306	0.286	0.091
		0.25	0.155	0.000	0.120	0.139	0.126	0.031
		0.50	0.053	0.008	0.053	0.057	0.053	0.020
		0.75	0.016	0.000	0.016	0.018	0.016	0.008
		0.90	0.004	0.000	0.009	0.009	0.009	0.005
Log Normal	*n* = 4	0.10	0.902	0.071	0.316	0.317	0.418	0.040
		0.25	0.758	0.040	0.177	0.144	0.369	0.015
		0.50	0.511	0.018	0.083	0.060	0.321	0.013
		0.75	0.257	0.004	0.055	0.040	0.188	0.066
		0.90	0.101	0.000	0.028	0.028	0.077	0.029
	*n* = 6	0.10	0.904	0.087	0.405	0.397	0.511	0.100
		0.25	0.753	0.051	0.212	0.203	0.415	0.046
		0.50	0.504	0.038	0.119	0.115	0.350	0.049
		0.75	0.253	0.004	0.056	0.055	0.199	0.103
		0.90	0.101	0.000	0.029	0.021	0.087	0.068
	*n* = 12	0.10	0.900	0.022	0.274	0.237	0.318	0.000
		0.25	0.750	0.016	0.106	0.092	0.192	0.000
		0.50	0.499	0.032	0.025	0.025	0.201	0.000
		0.75	0.249	0.000	0.022	0.017	0.156	0.015
		0.90	0.098	0.000	0.016	0.012	0.064	0.033
	*n* = 24	0.10	0.900	0.000	0.246	0.258	0.290	0.000
		0.25	0.750	0.000	0.096	0.103	0.252	0.000
		0.50	0.500	0.012	0.035	0.035	0.212	0.004
		0.75	0.250	0.000	0.021	0.019	0.136	0.014
		0.90	0.100	0.000	0.002	0.002	0.058	0.012
	*n* = 48	0.10	0.900	0.000	0.296	0.306	0.315	0.000
		0.25	0.750	0.008	0.139	0.133	0.190	0.000
		0.50	0.500	0.024	0.058	0.054	0.134	0.000
		0.75	0.249	0.000	0.019	0.021	0.109	0.000
		0.90	0.098	0.004	0.005	0.005	0.045	0.005
Mixed Normal	*n* = 4	0.10	0.894	0.000	0.274	0.303	0.397	0.045
		0.25	0.745	0.042	0.156	0.156	0.373	0.029
		0.50	0.488	0.016	0.100	0.086	0.344	0.014
		0.75	0.242	0.016	0.075	0.046	0.212	0.082
		0.90	0.094	0.000	0.039	0.032	0.089	0.054
	*n* = 6	0.10	0.899	0.021	0.338	0.309	0.460	0.000
		0.25	0.747	0.033	0.151	0.139	0.362	0.000
		0.50	0.489	0.020	0.077	0.077	0.347	0.013
		0.75	0.240	0.000	0.042	0.045	0.207	0.085
		0.90	0.094	0.000	0.020	0.016	0.094	0.062
	*n* = 12	0.10	0.898	0.020	0.390	0.383	0.484	0.020
		0.25	0.747	0.016	0.188	0.183	0.298	0.008
		0.50	0.497	0.004	0.088	0.088	0.318	0.012
		0.75	0.247	0.000	0.053	0.051	0.195	0.066
		0.90	0.097	0.000	0.022	0.016	0.082	0.050
	*n* = 24	0.10	0.900	0.000	0.342	0.333	0.390	0.000
		0.25	0.750	0.000	0.150	0.150	0.256	0.000
		0.50	0.501	0.064	0.064	0.067	0.288	0.004
		0.75	0.251	0.056	0.041	0.039	0.192	0.026
		0.90	0.100	0.023	0.018	0.018	0.085	0.035
	*n* = 48	0.10	0.899	0.000	0.254	0.265	0.333	0.000
		0.25	0.749	0.008	0.101	0.101	0.233	0.000
		0.50	0.500	0.004	0.027	0.023	0.231	0.000
		0.75	0.251	0.000	0.005	0.003	0.178	0.013
		0.90	0.100	0.000	0.007	0.002	0.084	0.022

*π*_1_: Proportion of differentially expressed genes.

TSBP: RBM Test statistic based permutation method.

TSBB: RBM Test statistic based bootstrap method.

PBP: RBM *p*-value based permutation method.

PBB: RBM *p*-value based bootstrap method.

In summary, the PBB method performs best when sample size is large with high sensitivity, specificity, and low false discovery rate, while the PM performs well on sensitivity and specificity across all sample sizes, except for a slightly higher false discovery rate when the proportion of differentially expressed genes is lower than 50%. The SAM method also performs well with high sensitivity, specificity, and low false discovery rate, except for low sensitivity when the proportion of differentially expressed genes is over 50% for all sample sizes.

### Simulation results from non-normally distributed data

In many cases, microarray data are not normally distributed and fail to be transformed to follow a normal distribution. Therefore, we explored the sensitivity, specificity, total rejection, and estimated false discovery rates of the PM, the SAM, and the RBMs for both log normally and mixed normally distributed data.

When data are log normal distributed (right skewed), the PM method shows the highest sensitivity of all methods under comparison across all sample sizes (Table 2). Both the SAM and the PBB methods show lower sensitivity than other RBM methods. The sensitivity of the PBB method improves as sample size increases. However, the SAM method exhibits a much lower sensitivity compared to all other methods when the proportion of differentially expressed genes is over 50%. When data follow a mixed normal distribution (left skewed and skinny tails), although the same pattern is observed for all methods, the PBB method shows improved sensitivity for all sample sizes. The SAM method still has the lowest sensitivity when the proportion of differentially expressed genes is greater than 50% for all sample sizes.

**Figure 3:**
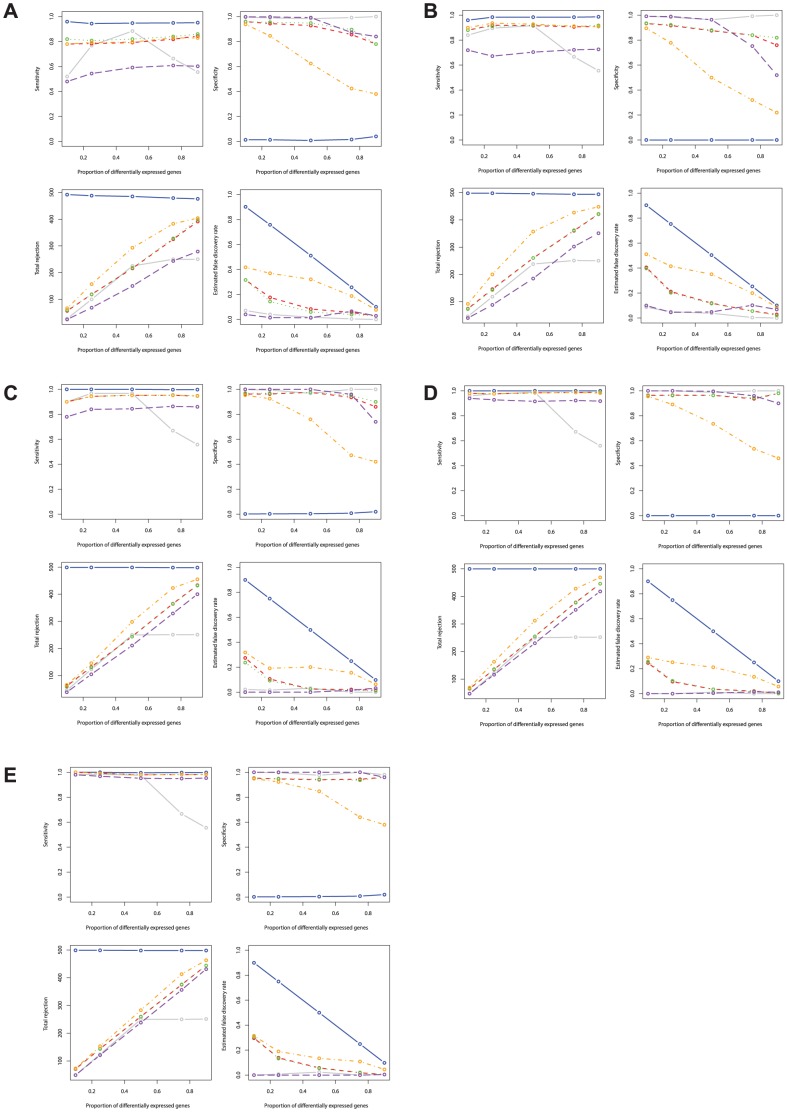
Sensitivity, specificity, total rejection, and estimated false discovery rate comparisons between the RBMs and the PM for lognormal distributed gene expression data. Blue: PM; Grey: SAM; Red: RBM test statistic based permutation method; Orange: RBM 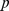-value based permutation method; Green: RBM test statistic based bootstrap method; Purple: RBM 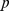-value based bootstrap method. Figure 2a: sample size *n* = 4 in each group; Figure 2b: sample size *n* = 6 in each group; Figure 2c: sample size *n* = 12 in each group; Figure 2d: sample size *n* = 24 in each group; Figure 2e: sample size *n* = 48 in each group.

**Figure 4:**
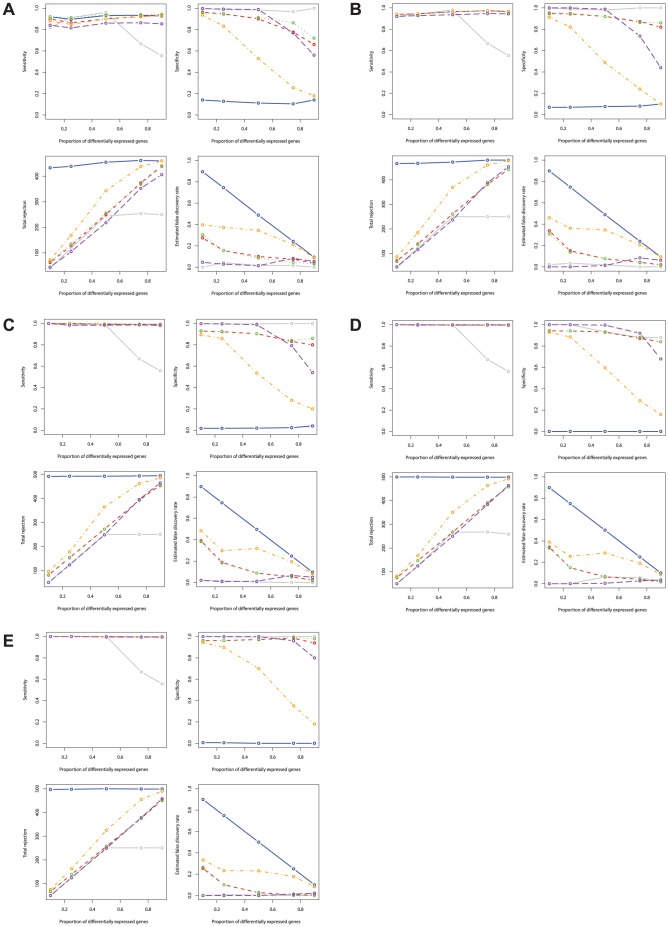
Sensitivity, specificity, total rejection, and estimated false discovery rate comparisons between the RBMs and the PM for mixed normal distributed gene expression data. Blue: PM; Grey: SAM; Red: RBM test statistic based permutation method; Orange: RBM 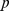-value based permutation method; Green: RBM test statistic based bootstrap method; Purple: RBM 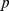-value based bootstrap method. Figure 2a: sample size *n* = 4 in each group; Figure 2b: sample size *n* = 6 in each group; Figure 2c: sample size *n* = 12 in each group; Figure 2d: sample size *n* = 24 in each group; Figure 2e: sample size *n* = 48 in each group.

The specificities of the PM method are significantly lower than all other methods regardless of sample size and proportion of differentially expressed genes for both log normal distributed data and mixed normal distributed data (Figure 3 and Figure 4). Both the SAM method and the RBMs have high specificity across all sample sizes, except that the PBP and the PBB methods have decreased specificity for all sample sizes when the proportion of differentially expressed genes is greater than 50% for both log normally distributed and mixed normally distributed data.

The number of total rejections for all methods also shows similar trends to sensitivity when data are either log normally or mixed normally distributed (Figure 3 and Figure 4). In contrast to normally distributed data, the PM method rejects almost all null hypotheses even when the proportion of differentially expressed genes are only 10% or 25% for all sample sizes. The number of total rejections for the RBMs is close to the true number of differentially expressed genes in the simulated data set. However, the SAM method rejects far less null hypotheses than the true number of non-true null hypotheses when the proportion of differentially expressed genes are over 50% across all sample sizes for either log normally or mixed normally distributed data.

The PM method's false discovery rate is the highest of all methods compared, for both log normally or mixed normally distributed data. The estimated false discovery rates for the PM method are significantly higher than all other methods especially for data characterized by a small proportion of differentially expressed genes such as 10% or 25% (Table 3). Of all methods, both the PBB and the SAM method show good control of false discovery rates at a 5% level, even when the proportion of differentially expressed genes is small and sample size is small.

In summary, the PBB method performs better than any other methods in terms of sensitivity, specificity and false discovery rate controls, when data are not normally distributed. The SAM method performs well, except for low sensitivity when the proportion of differentially expressed genes is over 50% across all sample sizes.

### Real data example

Preterm birth, which is defined as birth occurring before 37 weeks of gestation, can be harmful to the short-term and long-term health of the infant, and creates a large economic burden in the US. The estimated lower boundary of annual societal economic burden associated with preterm birth in the United States was in excess of $26.2 billion in 2005. A recent study on the role of DNA methylation in preterm birth indicates that DNA methylation is an epigenetic risk factor in preterm birth which may influence the risk of preterm birth, or result in changes predisposing a neonate to adult-onset diseases [20].

A methylation two-color microarray study (2012) was conducted at the University of Hawaii John A. Burns School of Medicine to identify placental DNA methylation loci associated with preterm delivery. The DNA of 9 women's placental tissue (originating from 4 premature births and 5 term deliveries) was analyzed. The gestational age distribution between the preterm delivery group and the term delivery group had been compared using the permutation test and no significant difference was found between this two groups (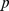-value = 0.556). Placental tissue was sampled from the decidual membrane rolls of previously formalin fixed samples. The decidual portion of the placenta predominantly consists of maternal tissue with a small percentage of fetal tissue. Using the Illumina® infinium bead chip, which utilizes bisulfite conversion across prespecified CpG sites across the genome, the percentage of DNA methylation in each of 485,577 loci was assessed. The “print-tip loess” normalization method was used to correct for within-array dye and spatial effects, while single channel quantile normalization was used to facilitate comparison between arrays.

**Figure 5:**
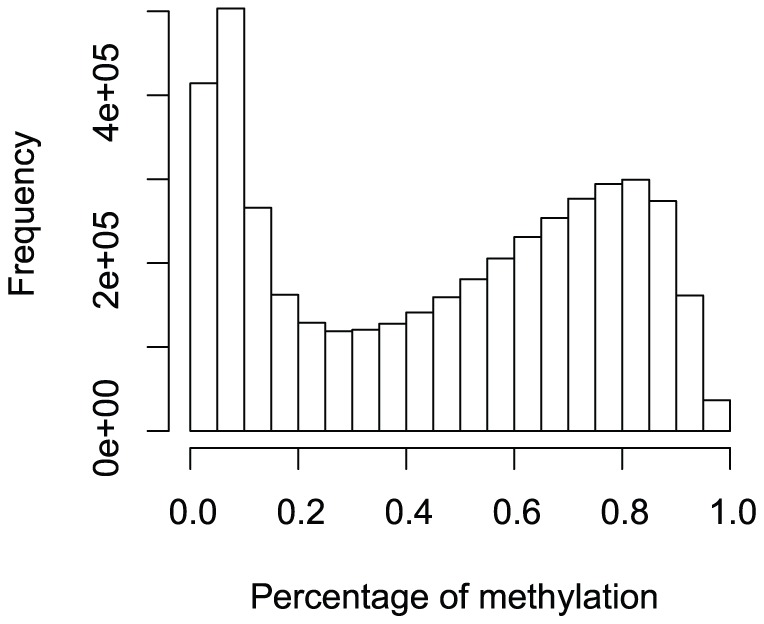
Histogram of percentage of methylation at 485,577 loci.

The percentage of methylation histogram at all loci shows that the distribution of the percentage data is not normally distributed (Figure 5). Both the RBMs, the SAM, and the PM were used to identify differentially methylated loci between premature births and term deliveries. Table 4 lists the total number of identified methylation loci by all six methods.

**Table 4:** Total discoveries comparison between the PM and the RBMs for 485577 loci.

Methods	PM	SAM	TSBP	TSBB	PBP	PBB
Total discoveries	476,554	0	34	351	19	304

TSBP: RBM Test statistic based permutation method.

TSBB: RBM Test statistic based bootstrap method.

PBP: RBM *p*-value based permutation method.

PBB: RBM *p*-value based bootstrap method.

According to the PM, over 98% of the loci are differentially methylated between placental tissues from 4 preterm women and 5 term women. This result indicates that the FDR is not well controlled by the PM. The SAM method rejected no loci for differential methylations between preterm deliveries and term deliveries which might due to the high conservativeness and low power of the SAM method. In contrast, the RBMs perform better than both the PM and the SAM methods, as it rejects a reasonable number of methylation loci. The number of rejections by the RBMs is comparable to the number of methylation loci identified by a recent DNA methylation microarray study [21], which identified 29 CpG sites among over 485,000 CpG sites associated with spontaneous preterm birth, independent of gestational ages.

## Discussion

The sensitivities, specificities, total rejections, and FDR controls were compared across the PM, the SAM, and the RBMs methods through simulation studies. The simulation results showed that this novel approach offers significantly higher specificity and lower false discovery rates compared to the PM method - for non-normally distributed data. This approach also offers higher statistical power than the SAM method when the proportion of significantly differentially expressed genes is large for both normally and non-normally distributed data. A real methylation microarray example was introduced to compare FDR controls across all methods. The RBMs rejected less than 1% of the methylation loci, which is comparable to the number of methylation loci identified by a recent study [21]. However, PM rejected over 98% of the methylation loci, and the SAM method rejected none of the methylation loci.

Our resampling-based empirical Bayes approach combined the resampling methods used by Westfall and Young [12] and Pollard and Van Der Laan [18] with the empirical Bayes method used by Smyth [2]. The robustness of the resampling methods to parametric distribution assumptions on test statistics was incorporated into the empirical Bayesian part of Smyth's method - which made the test statistics more robust to normal distribution assumptions, and less affected by either underestimated or overestimated sample variances compared to the ordinary test statistics used in the resampling procedures.

The PBB method and the SAM method always control the FDR at lower levels compared to other RBMs and the PM, for normally, lognormally, and mixed normally distributed data. The PM has a very large false discovery rate when microarray data are not normally distributed and the proportion of differentially expressed genes is small. FDR controls achieved by other RBMs (i.e., the PBP, the TSBP, and the TSBB methods) are significantly better than those achieved by the PM, but never as good as those achieved by the PBB method and the SAM method when data are not normally distributed. However, for normally distributed data, the performance of all other RBMs is similar to the PM.

Overall, the PBB method has the highest sensitivity, specificity, and the best FDR controls of all methods compared in this paper. Furthermore, the PBB method has much higher sensitivity than the SAM method when the proportion of differentially expressed genes is large, and much better FDR controls than the PM - especially when data are not normally distributed. The RBMs methods are computationally more intensive than Smyth's method as a result of the resampling approach; however, the computational efficiency of the RBMs methods could be greatly improved through a Bayesian algorithm that would reallocate more efficiently the number of resamples based on p-values [22].

A vexing issue with the Smyth's approach to microarrays analysis is its propensity to generate erroneous findings, especially when normality assumptions are violated. Our results show that the PBB method significantly improves microarray data analysis when normality assumptions are violated, and promotes accurate interpretation of findings from microarray studies. As Larsson pointed out, fold change criteria in the SAM method is problematic and can critically alter the conclusion of a study due to compositional changes of the control data set in the analysis [7]. As it turns out, our approach is not affected by the fold change threshold since no selection for the control data set is needed. Although the Resampling-based empirical Bayes Methods focus on two-color microarray data analysis, this novel approach could also be applied to single color oligonucletide microarrays, and generalized to next generation sequencing RNA-seq data analysis.
